# Postharvest Treatments on Sensorial and Biochemical Characteristics of *Begonia cucullata* Willd Edible Flowers

**DOI:** 10.3390/foods11101481

**Published:** 2022-05-19

**Authors:** Ilaria Marchioni, Isabella Taglieri, Rosanna Dimita, Barbara Ruffoni, Angela Zinnai, Francesca Venturi, Chiara Sanmartin, Laura Pistelli

**Affiliations:** 1 Department of Agriculture Food Environment, University of Pisa, Via del Borghetto 80, 56124 Pisa, Italy; ilaria.marchioni.16@gmail.com (I.M.); isabella.taglieri@for.unipi.it (I.T.); angela.zinnai@unipi.it (A.Z.); chiara.sanmartin@unipi.it (C.S.); laura.pistelli@unipi.it (L.P.); 2 Department of Biological and Environmental Science and Technologies (DiSTeBA), University of Salento, 7 Via Prov. le Lecce-Monteroni, 73100 Lecce, Italy; rosanna.dimita@unisalento.it; 3 Chambre d’Agriculture des Alpes-Maritimes (CREAM), MIN Fleurs 17 Box 85, CEDEX 3, 06296 Nice, France; 4 CREA—Centro di Ricerca Orticoltura e Florovivaismo, Corso Inglesi 508, 18038 Sanremo, Italy; barbara.ruffoni@crea.gov.it; 5 Interdepartmental Research Center, Nutraceuticals and Food for Health, University of Pisa, Via del Borghetto 80, 56124 Pisa, Italy

**Keywords:** dried flowers, hot-air drying temperature, freeze-drying technique, sensory evaluation, colorimetric analysis, sugars, polyphenol content, antioxidant activity

## Abstract

Edible flowers (EFs) are currently consumed as fresh products, but their shelf life can be extended by a suitable drying technique, avoiding the loss of visual quality and valuable nutraceutical properties. *Begonia cucullata* Willd is a common ornamental bedding plant, and its leaves and flowers are edible. In this work, *B. cucullata* red flowers were freeze-dried (FD) and hot-air dried (HAD) at different temperatures. To the best of our knowledge, our study is the first one comparing different drying methodologies and different temperatures involving sensory characterization of EFs; therefore, a codified method for the description of the sensory profile of both fresh and dried *B. cucullata* was developed and validated. Phytochemical analyses highlighted the better preservation of antioxidant compounds (polyphenols, flavonoids and anthocyanins) for flowers dried at 60–70 °C. Visual quality was strongly affected by the drying treatments; in particular the color of the HAD samples significantly turned darker, whereas the FD samples exhibited a marked loss of pigmentation. Although all drying conditions led to a reduction in the hedonic indices if compared with fresh flowers, the best results in terms of organoleptic properties were obtained when the drying temperature was set to 60 or 70 °C.

## 1. Introduction

Edible flowers (EFs) are traditionally consumed in many regions of the world, since their ornamental value, flavors and tastes have been appreciated for thousands of years [1]. *Begonia cucullata* Willd (syn. *Begonia semperflorens* Link & Otto; common English name: wax begonia) is one of the most common species [2], currently cultivated and consumed as EFs. This plant (Begoniaceae family) is native to South America, and it has naturalized elsewhere in tropics and subtropics [2,3]. *B. cucullata* has been produced as ornamental bedding plant for long time, and new hybrids and varieties were frequently created to meet the market requirements [4]. Currently, its use as EFs has been documented in various parts of the world [2,5]. Wax begonia flowers are characterized by several colors (scarlet, red, rose, white), a pleasant crispy texture and slightly lemon-like taste with a mild bitter aftertaste [1,2]. *B. cucullata* EFs are also a source of several secondary metabolites (mainly polyphenols, flavonoids and anthocyanins), useful to enrich a well-balanced diet with healthy molecules [6,7,8].

The consumption of freshly picked flowers is the most recommended choice to maintain brilliant colors, flavors and the highest content of antioxidant compounds, especially during the peak of blooming [2,9]. Despite the flowering period of *B. cucullata* is quite long (flowers bloom throughout the growing season until frost) [8], fresh flowers may not always be readily available on the market, since their shelf-life is rather short, even if stored at a low temperature (4–5 °C) [10], thus also limiting the geographic area of their supply chain. To date, few strategies have been evaluated for the improvement of the EFs storage, and only few studies have been carried out to determine the factors that limit their quality [11,12]. Drying is the common method used to extend EFs conservation, since it prevents microorganisms’ growth and enzymatic degradation [13]. This technique also reduces flowers weight and therefore the costs of transport and storage [13]. Temperature and time are crucial to maintain color, flower integrity, petal shape, bioactive compounds and sensory qualities as unaltered as possible [14]. Moreover, dried EFs have different uses from fresh flowers; they are consumed in teas, fine bakery or included in oil, vinegar and wine. In this way, dried EFs occupy their own market share and ensure sale opportunities independently from seasonality [15].

To date, the industry of EFs mainly operates with hot-air drying (HAD), even if other promising and innovative technologies have been experimentally tested recently (e.g., microwave drying, infrared freeze-drying and hybrid drying) [14,16]. HAD is a very cheap approach, easy to operate and to control. However, this technique requires long drying cycles, during which undesirable biochemical and nutritional changes can compromise the quality of the processed product [13,16]. To overcome these issues, heat-sensitive materials can be dried by more effective methods, such as freeze-drying (FD). This technique relies on low processing temperature, lack of oxygen and water sublimation to preserve the valuable characteristics of the fresh product [17]. Since high-energy consumption is required by the FD process, its application in food industry is limited, despite the remarkable quality of the freeze-dried products [17].

In this work, both HAD (50, 60, 70, 80 °C) and FD techniques were used to dry *B. cucullata* red flowers, and the effects are evaluated on their phytonutritional components and sensory qualities.

## 2. Materials and Methods

### 2.1. Chemicals

All chemicals used for the analyses, including methanol, NaOH, Na_2_CO_3_, AlCl_3,_ acetic acid, sulphuric acid, 2,4,6-trypyridyl-s-triazine (TPTZ), FeCl_3_, 2,2-diphenyl-1-picrylhydrazyl (DPPH) and anthrone were supplied by Sigma Aldrich (Milano, Italy) and Folin–Ciocalteu reagent by Merck (Darmstadt, Germany).

### 2.2. Plant Material and Cultivation

Young plants of *Begonia cucullata* Willd were donated by Lycée Horticole—Campus Vert d’Azur Antibes (Antibes, France), and immediately transplanted into 3-L pots at Chambre d’Agriculture des Alpes-Maritimes (CREAM, Nice, France, GPS: 43.668318 N, 7.204194 E), where they are grown in an unheated greenhouse until full bloom. Substrate composition and fertigation treatment were described in Marchioni et al. [18]. High quality flowers were harvested in the morning (between 8:00 and 10:00), weighed (FW) and then frozen at—80 °C (considered as fresh), freeze-dried or hot-air dried at different temperatures, as reported in the Postharvest treatments section.

### 2.3. Postharvest Treatments

Fresh flowers were weighted (FW) and vacuum freeze-dried (Labconco, Kansas City, MO, USA) for 48 h, or hot-air dried at 50, 60, 70 °C in Nutri Drya with constant ventilation (Dejelin, Courcelles, Belgium), and 80 °C (XBO32 Dryer, with adjustable ventilation, France Etuves, Chelles, France) for 20.5, 16, 11 and 3 h, respectively. The hot-air drying process was concluded when flowers dry weight (DW) remained unchanged over time, and the moisture was less than 10%. Weight loss percentage was calculated as follows: [(FW-DW) × 100]/FW. Hot-air drying was carried out at the Chambre d’Agriculture des Alpes-Maritimes (CREAM, Nice, France), whereas descriptive sensory evaluation and biochemical analyses on fresh, freeze-dried and hot-air dried flowers were performed at the Department of Agricultural, Food and Environment (DAFE), University of Pisa.

### 2.4. Color Parameters

The color of the different flowers was measured according to the Commission Internationale de l’Eclairage CIE L*a*b* Color System by means of a tristimulus colorimeter (Eoptis, Mod. CLM-196 Benchtop, Trento, Italy). The analysis was performed on flowers lying on an area of approximately 24 cm^2^ and each sample was analyzed in triplicate. The color was defined on the base of the chromatic coordinates [19], lightness (L*), green-red (a*) and blue-yellow components (b*). The Chroma value C*, which is an expression of color saturation, was also used to evaluate the color, and calculated by the relation:

(1) $$C^{*}=\sqrt{a^{*}{}^{2}+b^{*}{}^{2}}$$

The color difference among samples was expressed as $\Delta E_{\mathrm{ab}}^{*}$:

(2) $$\Delta E_{\mathrm{ab}}^{*}=\sqrt{\Delta L^{*}{}^{2}+\Delta a^{*}{}^{2}+\Delta b^{*}{}^{2}}$$

### 2.5. Descriptive Sensory Evaluation

**Figure 1:**
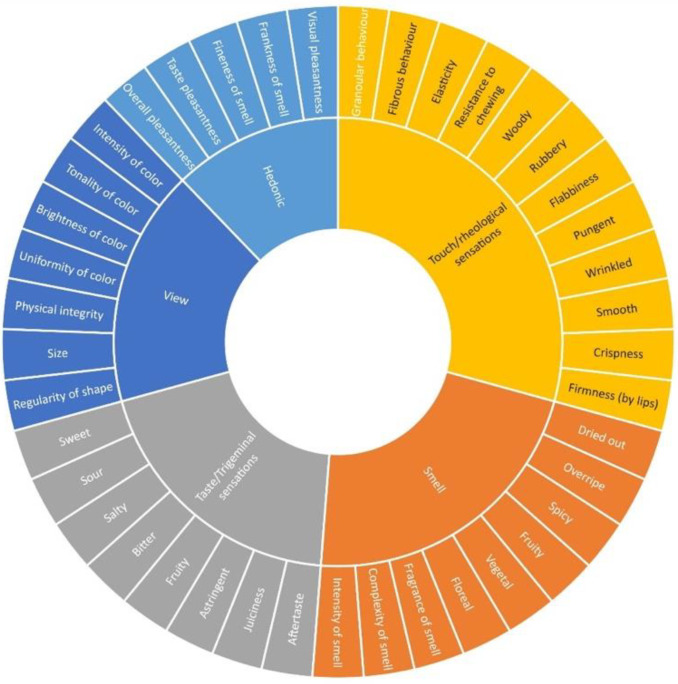
Sensory wheel of flower analysis (XLSTAT ver. 1 April 2019).

Sensory profiles of the flowers were determined by a descriptive analysis by a panel of trained assessors (10 assessors, 6 females and 4 males, aged between 23 and 60 years) from the “expert panel” of the DAFE of the University of Pisa. The DAFE internal procedure for assessor selection and the training was applied as previously reported [20]. A specific training session was organized before the beginning of the specific tasting sessions, with the aim of defining the specific method of the sensory evaluation of flowers. All of the trained panelists were firstly involved in a consensus panel specifically aimed at generating descriptors and their definitions. A final set of 41 descriptive parameters, including both quantitative and hedonic attributes, was individuated by agreement among panelists, and an innovative sensory wheel specific for the tasting of flowers was developed (Figure 1).

### 2.6. Biochemical Analyses

Total polyphenols, flavonoids and anthocyanins content, as well as the flowers’ antioxidant activity, the 2,2-diphenyl-1-picrylhydrazyl radical (DPPH) assay and Ferric ion Reducing Antioxidant Power (FRAP) assays, were determined prior to extraction of fresh (150 mg) and dried (20 mg) flowers in 2 mL of 70% (*v*/*v*) methanol solution, as already described [18]. The Folin-Ciocalteu method, according to Singleton and Rossi [21], was followed to quantify the flowers’ total polyphenolic content (TPC). Sample absorbance was read at 765 nm (Ultraviolet-Visible spectrophotometer, SHIMADZU UV-1800, Shimadzu Corp., Kyoto, Japan), and the results were expressed as mg gallic acid equivalent (GAE) per g FW or DW. Total flavonoids content (TFC) was determined as reported in Kim et al. [22]. The absorbance was read at 510 nm and the concentration was expressed as mg of (+)-catechin equivalents (CE) per weight of samples. The determination of the DPPH scavenging activity was performed according to Brand-Williams et al. [23], and the data were expressed as mmol Trolox Equivalent Antioxidant Capacity (TEAC) for weight of samples. The FRAP assay was performed according to Szôllôsi and Szôllôsi Varga [24], and the results were reported as mmol FeSO_4_/g FW or DW. The total soluble sugars (TSS) were extracted as reported in Das et al. [25], starting from 150 or 20 mg of fresh and dried flowers, respectively. TSS were spectrophotometrically estimated as described in Marchioni et al. [18], and data were reported as mg glucose per g FW or DW.

Total acidity (TTA) was determined by acid–base titration, according to Li et al. [26], with some modifications. Briefly, 50 mg of sample was mixed in water (solid/liquid extraction, ratio 2:1, *w*/*v*) and sonicated for 15 min at room temperature. After paper filtering, the extract was then titrated with NaOH 0.01 N, using 1% phenolphthalein as an indicator and the TTA was expressed as meq of citric acid/g of sample. The pH of the extract was determined with a pH-meter (pH 80+ DHS, XS Instrument, Modena, Italy).

### 2.7. Statistical Analysis

Biochemical results were statistically analyzed by one-way analysis of variance (ANOVA) (Past3, version 3.15), using either Tukey Honestly Significant Difference (HSD) or the Mann–Whitney U test according to the variance homogeneity (Levene test), with a cut-off significance of *p* < 0.05 (letters).

Two-way ANOVA and choosing samples and panelists as main factors after processing the results by Big Sensory Soft 2.0 (ver. 2018) was applied for sensory data.

## 3. Results and Discussion

### 3.1. Colorimetric Analysis

**Figure 2:**
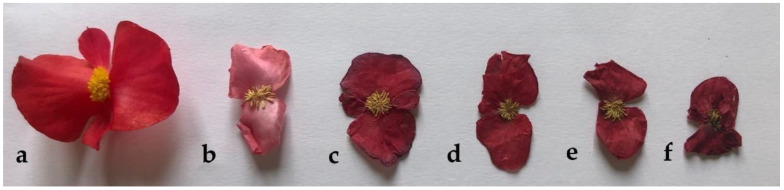
Visual quality of *B. cucullata* flowers. Fresh (**a**), freeze-dried (FD; **b**), hot-air dried (HAD) 50 °C (**c**), HAD 60 °C (**d**), HAD 70 °C (**e**), HAD 80 °C (**f**).

The color represents one of the most important characteristics with respect to the postharvest handling of EFs, since it affects the enjoyment of eating [27]. In particular, red flowers are able to stimulate appetite [28]; thus, the proper postharvest treatment should maintain this feature as much as possible to preserve *B. cucullata* attractiveness. The flowers appearance after the different treatments are shown in Figure 2.

As observed by Zhang et al. [29], the color of the samples significantly changed because of the drying process, turning darker, with the exception of FD samples, in which a loss of pigmentation occurred (Figure 2 and Table 1).

Moreover, FD flowers showed an increase in brightness (L* value, Table 1), contrary to what observed in *Tagetes erecta* [27], red rose and carnation [30]. Redness parameter a* appeared significantly reduced in all dried samples, if compared with fresh flowers. This is true especially for the sample treated at 80 °C, showing how the highest drying temperature led to more serious color degradation. In addition to a* values, HAD treatments also induced a reduction in the bluish parameter, confirming what observed by Siriamornpun et al. [27] on *T. erecta* flowers. These authors suggest that the reduction of those color coordinates was probably due to both non-enzymatic browning reactions and the destruction of pigments in the petals.

Chroma significantly changed as a consequence of the drying processes carried out, taking on a reduced saturation and an opaque appearance with the increasing temperature applied (Table 1).

As outlined by the distance between the chromatic coordinates ($\Delta E_{\mathrm{ab}}^{*}$) (Table 2), all samples could be visibly discriminated in color when compared to each other, in accordance with the above-discussed results. Starting from recent findings on this topic for different EFs [31,32], in the future, our investigations could be made on the pretreatments of flowers before drying to reduce the color depletion compared to the fresh ones.

### 3.2. Descriptive Sensory Evaluation

Organoleptic performance, flavor and overall impression are pivotal to evaluate the quality of flowers intended for culinary uses [33]. Despite the favorable results on their content of bioactive compounds, there is little information on consumers organoleptic preference of EFs, and their sensory characterization has been limited only to a small amount of species so far in the literature [33,34]. In addition, whereas the use of pre-treatments, such as drying, as a preservation technique to extend EFs shelf life, can deeply affect their overall quality, and, as a consequence, their acceptability [31,32], to the best of our knowledge, no data are available about the impact of different drying techniques on sensory expression of EFs.

**Figure 3:**
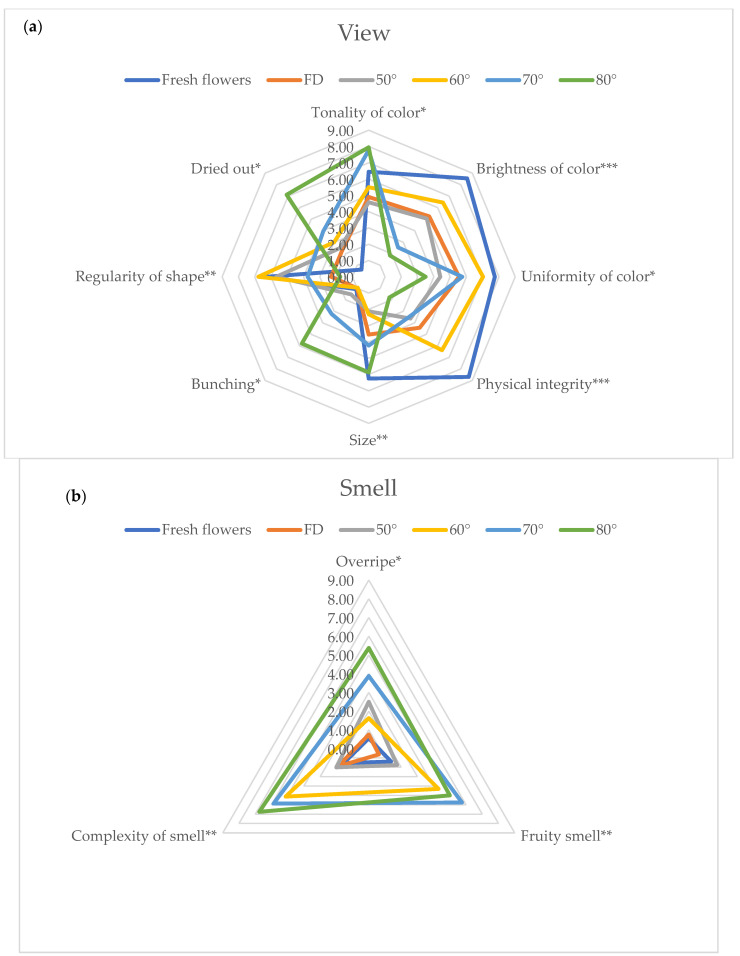

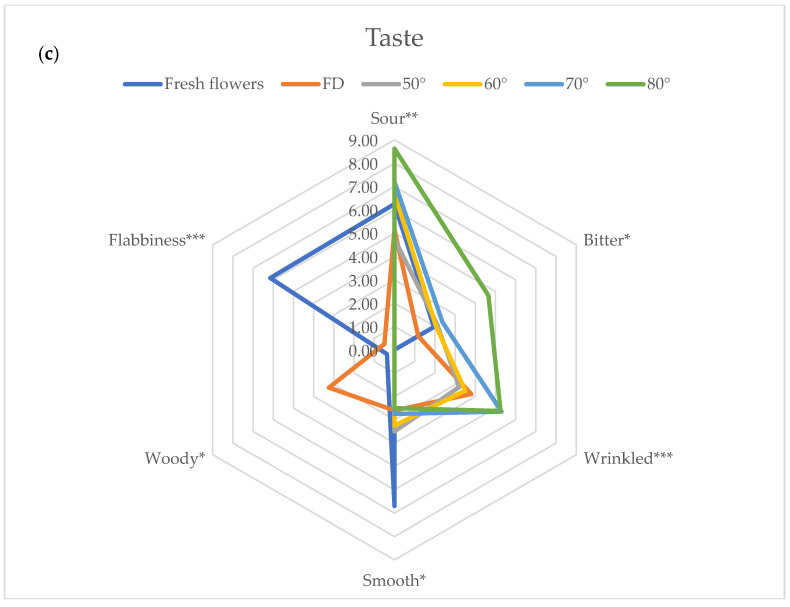
Flowers’ sensory profile related to View (**a**), Smell (**b**) and Taste (**c**). The figures only report the parameters that showed statistically significant differences. Significance level *** *p* < 0.001, ** *p* < 0.01; * *p* < 0.05. Abbreviation: FD—freeze-dried.

During panel tests all the parameters selected and reported in Figure 1 were addressed and evaluated by the judges. For clarity of exposure, in Figure 3a–c, only sensorial parameters that showed significant differences among treatments were reported and further discussed. At a visual level, the higher the temperature utilized for drying, the higher the changes were, as reported by panelists, with a significant reduction of brightness and uniformity of color together with the physical integrity, whereas the bunching appears significantly increased when the drying temperature reached 70 and 80 °C. When the drying temperature reaches and exceeds 60 °C, there is a significant increase in Complexity of smell together with the characters of Fruity and Overripe. On the other hand, the Sour and Bitter tastes increased significantly when the drying temperature reached 80 °C.

**Figure 4:**
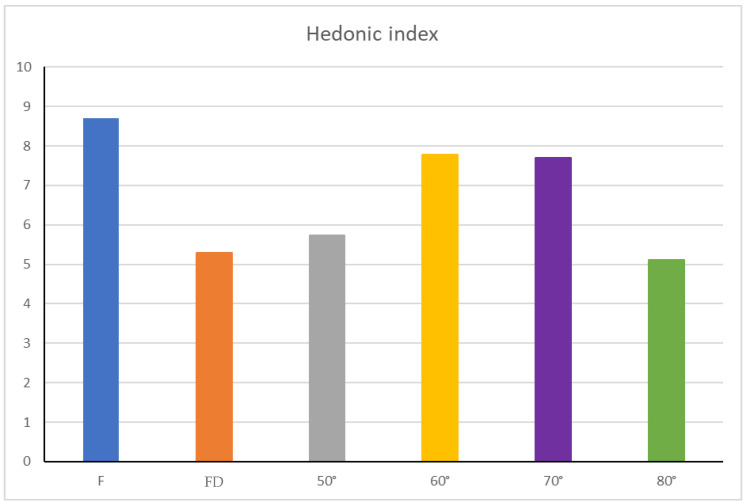
Overall hedonic indices of dried flowers. Abbreviation: F—fresh flowers; FD—freeze-dried.

When a new technical process in food production is explored, the level of hedonic quality, expressed by the obtained products, is fundamental in determining its consumer acceptability [35]. Therefore, some hedonic parameters related to view, smell, taste and overall pleasantness were also evaluated (Figure 4) to obtain preliminary information about the impact of the treatments used on the organoleptic acceptance of the different samples. In this context, Benvenuti et al. [34] showed the dependence on different personal taste from the evaluation of *B. cucullata*, due to its particular acidic taste, even comparable to lemon taste perception. Through the overall hedonic index (Figure 4), calculated by the means of the values attributed during panel tests to each hedonic parameter, and converted on a scale from 0 to 10 (Equation (3), it was possible to evaluate the whole organoleptic quality of all the flowers’ objects of the research.

(3) Overall hedonic index=MEAN[Hedonic indices]∗1,11 

As shown in Figure 4, fresh flowers had the highest overall hedonic index, suggesting their potential greater consumers’ acceptability over dried flowers. Nevertheless, even if all drying conditions led to a reduction in the hedonic indices of flowers, the best results, in terms of organoleptic properties, were obtained when the drying temperature was set up at 60 or 70 °C.

To the best of our knowledge, very few studies were performed on EFs descriptive sensory evaluation, involving only fresh flowers [13,33,34,36]. Earlier reports were mainly consumers’ preference surveys, in which flowers’ overall quality and colors were evaluated for some well-known species, such as pansy (*Viola* × *wittrockiana* Gams) [37], viola (*Viola tricolor* L.), borage (*Borago officinalis* L.) and nasturtium (*Nasturtium officinalis* L.) [28,38]. Until now, only one work has compared EFs postharvest treatment, also from a sensory point of view, evaluating gamma-irradiated *Bauhinia variegata* L. flowers with three different doses [39]. Therefore, our study is the first one comparing different drying methodologies and different temperatures involving sensory characterization of EFs.

### 3.3. Biochemical Analyses

In recent time, worldwide consumers’ demand for EFs increased due to their phytochemical content with healthy properties [9]. A primary goal of postharvest treatment (e.g., drying methods) is the maintenance of the highest phytonutritional content. Therefore, some primary and secondary metabolites were quantified in red *B. cucullata* fresh, freeze-dried (FD) and hot-air dried (HAD) flowers (Table 3).

Fresh flowers are characterized by a good amount of phenolic compounds and antioxidant activity, despite their high water content. In fact, regardless to the drying methods and the temperature applied, flower weight loss was higher than 90% (data not shown). This percentage was comparable in all treatments, varying between 93.2 (HAD 70 °C) and 95.6% (HAD 80 °C) (data not shown). Total phenolics content (TPC) was comparable to Chensom et al. [7] and Traversari et al. [40], even if we highlighted a higher antioxidant activity (3.43 mmol TEAC/g FW). Despite the same color, total anthocyanins were significantly higher in our samples, if compared with the ones of Traversari et al. [40].

Regarding dried flowers, the highest content of total polyphenolic compounds was detected in FD and HAD 50 °C samples, followed by HAD 60 and 70 °C. The highest tested temperature led to the most significant decrease of these metabolites. A similar trend was also observed for the total flavonoids content (TFC), even if slight differences between FD and HAD flowers were detected. Similarly to TPC, TFC decreased significantly with increasing temperature, reaching the lowest amount at 70 and 80 °C (Table 3). In the literature, several works highlighted the reduction of TPC and TFC when flowers were dried with high temperatures, and FD is identified as a better drying method than HAD [14,41,42,43]. Nevertheless, our results showed a good amount of total phenolics still guaranteed up to 70 °C, in agreement with that observed in *Camelia sinensis* flowers (comparable TPC between FD and HAD 60 °C samples) [43]. This is probably also due to an inactivation of polyphenol oxidases (PPO) that occurred at temperatures higher than 50 °C, which prevents their oxidation [42]. This hypothesis was supported also by Tan et al., 2015 [44] and Loh and Lim, 2017 [45], who reported very low residual PPO activity (<3%) in *Morus alba* and *Persea americana* leaves, respectively, after drying treatment at 50 °C for few hours (4–5 h). The study of the activity of enzymes that oxidize phenolic compounds is beyond the aims of this work. Further investigation on the topic should be performed to elucidate the physiological responses of the drying process in *B. cucullata* flowers, also evaluating enzymes stability at different temperatures. Peroxidase (POD) should be also taken into account, since it is considered a most heat-stable vegetable enzyme, able to oxidize phenolic compounds, also leading to negative flavor changes during storage [46]. Nevertheless, the drying temperature tested in our work should be enough to significantly decrease POD activity, on the basis of the literature data [47].

In addition to drying time and temperature, water activity (WA) should be also taken into account for further investigation, since those parameters could synergistically affect enzymatic inactivation during the drying process [48].

On the other hand, TPC reduction observed in HAD *B. cucullata* flower at 80 °C was probably linked to their degradation at high temperatures [49].

The highest amount of total monomeric anthocyanins was detected in HAD 60 and 70 °C flowers, followed by the ones dried at 50 °C, 80 °C and FD. Despite anthocyanins being heat sensible, the shorter time of exposure at 70° C than 50 °C (respectively 11 and 20.5 h) causes less damage to this class of metabolites. Similar behavior was observed in lilac *Bletilla striata* flowers [50]. Surprisingly, 80°C and FD shared the lowest content of anthocyanins. A temperature of 80 °C could be too high for anthocyanins, whereas FD treatment could led to higher pH values. To validate this hypothesis, further investigations are required to explain the loss of color in FD begonia flowers (Figure 2) (e.g., identification of flowers red pigment and their kinetic of degradation).

FD is a drying technique known to maintain an unaltered and high quality of food products, able to prevent chemical decomposition due to its low processing temperature and lack of oxygen [17]. FD begonia flowers showed the most remarkable radical scavenging activity (DPPH assay), a parameter which was almost halved in HAD 80 °C samples. These results were confirmed by the ferric ion reducing antioxidant power (FRAP) assay, where the data showed the same trend. Among HAD samples, no substantial differences were highlighted in begonia flowers dried from 50 to 70 °C.

Taken together, HAD flowers at 60 and 70 °C could be a fairly fast drying method, that would allow to obtain *B. cucullata* dried flowers with high amount of phenolic compounds, anthocyanins included, and good antioxidant activity.

The highest amount of total soluble sugars and acidity were quantified in HAD flowers at 70 and 80 °C, whereas a progressive decrease was observed for both parameters at lower temperatures (Table 3). With regards to sugars, contrasting data are present in the literature. Our results are similar to those obtained in chrysanthemum flowers, with a higher amount of soluble sugars detected in oven dried samples at 65 and 75 °C than in those oven dried at 55 °C [51]. Contrary to our observation, Park et al. [48] and Marchioni et al. [18] reported that 50 °C is the proper temperature to retain carbohydrates in *Agastache rugosa* and *A. aurantiaca* flowers, respectively. Interestingly, *A. rugosa* HAD flowers at 50 °C showed the highest content of sucrose [52], which is the soluble sugar mainly perceived as sweet to the human palate [53]. Despite no specific compound being identified in our work, no significant differences on sweet taste were observed in begonia flowers at any temperature tested.

To the best of our knowledge, very few data are available on HAD flowers and titratable acidity (TTA). Fernandes et al. [54] investigated the TTA in freeze-dried and HAD (50 °C) *Centaurea cyanus* flowers. The results cannot be easily compared with ours, since completely different drying cycles were applied (a few hours vs. 20.5 h). On the other hand, Park et al. [52] observed that oven-dried flowers at 80 °C were significantly higher in tricarboxilic acid cycle (TCA) intermediates (citric acid, fumaric acid and succinic acid) than FD and other oven-dried samples. Sour intensity is mainly determined by the presence of organic acids, such as citric acid [49], and our results could be in agreement with those observations. In fact, we observed higher TTA in flowers treated at the highest temperatures (70 and 80 °C), together with the perception of sour taste (Figure 3c and Table 3).

As it is known in the literature, phenolic compounds, mainly flavonoids, are responsible for the bitterness of plants product [55], and these bioactive compounds showed dramatic changes at the beginning of the drying process, probably due to unsteady states of heat and mass transfer simultaneously [43]. Fernandes et al. [56] showed a clear correlation between flowers tastes and bioactive compounds in their petals. Drying cycles of different lengths should be tested in future works, in order to check and avoid the molecules responsible for the bitter taste.

## 4. Conclusions

The consumption of EFs such as *B. cucullata* could be a valuable solution to enrich diets with bioactive compounds (i.e., polyphenols, flavonoids and anthocyanins). It is indeed well known that following a well-balanced diet protects against malnutrition in all its forms, as well as noncommunicable diseases, such as diabetes, heart disease, stroke and cancer. Fresh EFs bring the highest content of healthy molecules; however, they have a reduced shelf life, and thus they are available on the market only for short periods and in a limited distribution area close to the producing site. Therefore, the main purpose of the research was to investigate the effect of different drying procedures (freeze-drying and hot-air drying at 50, 60, 70, 80 °C) in order to select the best working conditions suitable to maximally improve the EFs stability, and in the meantime, avoiding or at least reducing the loss of visual quality, sensory and nutraceutical properties, without the addition of preservatives or other additives. Moreover, our study is the first one comparing different drying methodologies and different temperatures involving sensory characterization of EFs; therefore, a codified method for the description of the sensory profile of both fresh and dried *B. cucullata* was developed and validated.

Visual quality was strongly affected by the drying treatments, in particular the color of the HAD samples significantly turned darker, whereas the FD samples exhibited a marked loss of pigmentation.

As expected, most of sensorial parameters as well as the overall organoleptic profile are deeply affected by the drying method. However, in the experimental conditions adopted, the higher overall organoleptic quality in dried flowers was observed when the drying temperature was set up at 60 or 70 °C.

To conclude, the consumption of dried edible flowers leads to developing new distribution channels, and the description of sensorial features, along with the preservation of phytochemical compounds, are required to define their quality. Biochemical results suggest that medium-high temperatures could be used to obtain dried *B. cucullata* flowers rich in molecules with healthy value. According to our results, *B. cucullata* flowers showed a good conservation of sensorial and phytochemical features at drying temperatures of 60 and 70 °C.

## Figures and Tables

**Table 1:** CIE L*a*b* color parameters of the *B. cucullata* flowers subjected to different postharvest treatments. Abbreviation: FD—freeze-dried; HAD—hot-air dried.

	*p*-Value ^1^	Fresh Flowers	FD	HAD 50 °C	HAD 60 °C	HAD 70 °C	HAD 80 °C
L*	***	34.64 ^b^	46.85 ^a^	32.3 ^bc^	32.48 ^bc^	32.28 ^c^	28.49 ^d^
a*	***	44.43 ^a^	38.19 ^b^	31.87 ^c^	33.67 ^c^	35.81 ^bc^	22.78 ^d^
b*	***	17.97 ^a^	6.99 ^cd^	3.45 ^e^	11.64 ^b^	5.68 ^d^	7.88 ^c^
C*	***	47.93 ^a^	38.83 ^b^	32.08 ^c^	35.62 ^bc^	36.28 ^bc^	24.15 ^d^

^1^ Significance level *** *p* < 0.001, ns: not significant (*p* > 0.05). L* = lightness a* = green-red components; b* = blue-yellow components; C* = Chroma. In the same column, different letters indicate significant differences among samples.

**Table 2:** CIE L*a*b* color differences ($\Delta E_{\mathrm{ab}}^{*}$ ) among flowers samples.

$\Delta E_{\mathrm{ab}}^{*}$	Fresh Flowers	FD	HAD 50 °C	HAD 60 °C	HAD 70 °C	HAD 80 °C
Fresh flowers		17.56	19.33	12.67	15.19	24.67
FD			16.22	15.76	14.82	23.99
HAD 50 °C				8.39	4.53	10.82
HAD 60 °C					6.34	12.19
HAD 70 °C						13.75
HAD 80 °C						

**Table 3:** Determination of anthocyanins, total phenolics, total flavonoids, antioxidant activity (DPPH and FRAP assay), total soluble sugars (TSS) and pH and total acidity (TTA) in *B. cucullata* flowers subjected to different postharvest treatments. Data are expressed as mean ± standard deviation (*n* = 3). Within drying treatments, different letters mean significant differences using Tukey HSD or the Mann–Whitney U tests, with a cut-off significance of *p* < 0.05.

	Fresh Flowers *	FD	HAD 50 °C	HAD 60 °C	HAD 70 °C	HAD 80 °C
Total anthocyanins (mg Cy3G/g DW)	0.28 ± 0.02	5.47 ± 0.20 ^c^	6.34 ± 0.09 ^b^	8.59 ± 0.47 ^a^	9.35 ± 0.40 ^a^	4.47 ± 0.49 ^c^
Total phenolics (TPC) (mg GAE/g DW)	1.66 ± 0.26	55.90 ± 4.12 ^a^	55.36 ± 1.82 ^a^	44.36 ± 2.08 ^ab^	48.25 ± 0.93 ^ab^	39.38 ± 0.31 ^b^
Total flavonoids (TFC) (mg CE/g DW)	0.64 ± 0.06	27.18 ± 3.13 ^a^	25.05 ± 1.14 ^ab^	19.91 ± 1.37 ^ab^	17.09 ± 0.42 ^b^	12.73 ± 1.40 ^b^
Radical scavenging activity (DPPH assay) (mmol TEAC/g DW)	3.43 ± 0.28	191.06 ± 3.46 ^a^	131.48 ± 0.63 ^b^	127.78 ± 4.18 ^b^	128.41 ± 1.75 ^b^	105.47 ± 0.35 ^c^
Ferric ion reducing antioxidant power (FRAP assay) (mmol FeSO_4_/g DW)	13.33 ± 1.71	510.80 ± 34.18 ^a^	437.06 ± 18.82 ^b^	390.33 ± 4.92 ^bc^	433.53 ± 15.16 ^b^	349.48 ± 10.96 ^c^
Total soluble sugars (mg GLU/g DW)	6.96 ± 0.03	125.70 ± 7.06 ^b^	109.15 ± 2.11 ^b^	112.70 ± 3.38 ^b^	171.06 ± 0.49 ^a^	171.25 ± 5.19 ^a^
pH	3.36 ± 0.15	3.39 ± 0.08 ^a^	3.24 ± 0.01 ^ab^	2.99 ± 0.08 ^bc^	2.92 ± 0.01 ^c^	2.95 ± 0.06 ^c^
Total acidity (TTA) (mEq citric acid/g DW)	0.07 ± 0.01	1.63 ± 0.26 ^c^	1.69 ± 0.32 ^c^	2.69 ± 0.10 ^b^	3.19 ± 0.16 ^ab^	3.47 ± 0.07 ^a^

* Fresh flower’s data are reported as gFW^−1^. (fresh weight)^.^ Abbreviation: FD = freeze-dried; HAD = hot-air dried; DW = dried weight; GLU = glucose; CE = (+)-catechin equivalents; GAE: gallic acid equivalents.

## Data Availability

Data available on request from the authors.
